# Construction of a specialized nurse training Indicator system for cardiac intensive care unit using the Delphi method

**DOI:** 10.3389/fmed.2026.1932888

**Published:** 2026-09-07

**Authors:** Zhiyun Yang, Jing Huang, Songmei Xiao, Yuxiao Sun, Shiyu Li, Feng Li, Hongping Xu, Yuping Zhang, Huili He, Lili Ma

**Affiliations:** 1 CICU, Shanghai East Hospital, School of Medicine, Tongji University, Shanghai, China; 2 Department of Nursing, Shanghai East Hospital, School of Medicine, Tongji University, Shanghai, China; 3 Cardiacvascular Disease Ward, Shanghai East Hospital, School of Medicine, Tongji University, Shanghai, China

**Keywords:** cardiac intensive care unit, competency, Delphi method, specialized nurses, training system

## Abstract

**Aim:**

This study aims to establish a standardized training indicator system for cardiac Intensive Care Unit (CICU) specialized nurses based on competency theory, so as to provide practical guidance for improving their core competencies and guaranteeing high-quality care for critically ill patients.

**Methods:**

The training indicator system was developed through literature review, semi-structured qualitative interviews and Delphi method. Literature review was conducted to extract competency dimensions and indicators covering professional knowledge, professional skills, professional competence and practical competence; semi-structured qualitative interviews with clinical and educational specialists were further carried out to develop an initial training indicator system with 4 primary, 19 secondary and 60 tertiary indicators. Followed by two rounds of Delphi consultation, 23 experts specializing in CICU Medicine, CICU nursing management, CICU clinical nursing, and nursing education were invited to participate. Indicators were retained when importance score >3.50 and coefficient of variation <0.25. The Analytic Hierarchy Process (AHP) was used to determine the weights of indicators at different levels.

**Results:**

Both two rounds of expert surveys achieved a 100% response rate, with expert authority coefficients of 0.884 and 0.894, respectively. The Kendall’s ranged from 0.241 to 0.247 (*p* < 0.001), and the coefficient of variation ranged from 0.070 to 0.131. The final version of the training indicator system includes 4 first-level indicators, 19 secondary-level indicators, and 63 tertiary-level indicators. All indicators obtained importance scores above 4.47 with reasonable weight distribution.

**Conclusion:**

This competency-oriented CICU specialized nurse training indicator system possesses favorable scientificity and operability, and can serve as a reference for standardized professional development.

## 1 Introduction

The Cardiac Intensive Care Unit (CICU) serves as the primary facility for the treatment of patients with critical cardiovascular diseases. It is tasked with the diagnosis and management of a range of severe cardiovascular conditions, including acute myocardial infarction, severe heart failure, and cardiogenic shock in high-risk patients (1, 2). These patients exhibit highly variable clinical conditions and require complex treatment approaches, which imposes stringent demands on nurses’ professional competence (3). The National Nursing Development Plan (2021–2025) explicitly states that training programs for specialized nurses should be guided by job requirements and centered on professional competency (4). With the rapid updates in cardiovascular interventional therapy, extracorporeal membrane oxygenation (ECMO), and pulse contour cardiac output (PiCCO) monitoring technology, the gap between existing nursing training content and actual clinical demands has become increasingly prominent.

At present, the domestic construction of critical care nurse training systems mostly targets general ICU nurses, while specialized competency frameworks and matched standardized training schemes exclusive to CICU remain insufficient (5, 6). The existing CICU off-the-job and on-the-job training work faces prominent practical dilemmas: curriculum modules are fragmented, training content overlooks unique cardiovascular critical care operation demands, unified quantitative assessment indicators are absent, resulting in obvious individual differences in nurses’ emergency disposal, multidisciplinary collaboration and clinical decision-making ability, which cannot support high-quality standardized cardiovascular critical care services (7–9). Establishing a CICU specialist nurse training system with clear competency orientation and standardized content is an urgent need to ensure the safety of cardiovascular critical care patients and improve the quality of specialist nursing.

Competency” refers to the abilities held by a specific professional when completing a particular task or action; it also requires relevant specialized training and corresponding educational background to successfully accomplish the task (10). Professional competency refers to the comprehensive set of knowledge, skills, abilities, and characteristics within an organization that enable employees to perform effectively in their roles and achieve outstanding work performance (11, 12). The professional competency theory emphasizes the “alignment between abilities and job requirements” (13). By defining core elements such as knowledge, skills, and attitudes required for specific positions, it establishes a systematic training framework that has been widely applied in specialized fields such as critical care nursing and emergency nursing (14). However, due to the unique characteristics of CICU patients—including acute onset, rapid progression, high mortality rates, and complex complications—specialized nursing care demands significantly higher competencies from nurses compared to conventional intensive care nursing (15–17). These include specialized cardiovascular assessment, prompt management of critical cardiovascular conditions, and proficiency in operating dedicated cardiovascular medical devices (15–17).

Existing studies fail to systematically sort out the core competency demands of CICU specialized nurses, nor do they establish a targeted, layered training indicator system that integrates curriculum design, skills training and performance evaluation. Therefore, the core research question of this study is: how to construct a competency training indicator system for CICU specialized nurses based on the actual clinical demands, and provide a scientific and operable reference for the training, assessment and professional development of CICU specialized nurses in China. This provides a theoretical basis and practical guidance for standardizing the training of CCU specialist nurses, improving the quality of cardiovascular critical care, and guaranteeing optimal treatment outcomes for critically ill patients.

## 2 Materials and methods

### 2.1 Formation of research team

The research team consisted of seven members: one chief physician specialized in CICU, two CICU specialized nurses, one chief nurse, one associate chief nurse, and two senior clinical nurses. All team members held at least a bachelor’s degree, with more than 10 years of cumulative CICU clinical, nursing teaching and research experience. The team was responsible for conducting literature search and analysis, developing the Delphi consultation questionnaire, selecting experts for the correspondence survey, and organizing, analyzing, and summarizing expert opinions.

This study was approved by the Ethical Committee of Shanghai East Hospital (Approval No. 2025YS-075). During the Delphi procedure, we strictly adhered to ethical research standards to ensure that the rights and interests of all participating experts were protected. After gaining a full understanding of purpose and design about this study, experts voluntarily decided whether to participate and signed informed consent forms. We maintained strict confidentiality of all expert data and opinions, and all these materials were used solely for the purpose of this research. We also applied anonymization to protect the experts’ privacy.

### 2.2 Draft version of competency-based CICU specialized nurse training indicator system

#### 2.2.1 Literature review

To identify core competency dimensions of CICU nurses, we conducted literature retrieval in Chinese databases (CNKI, Wanfang, VIP) and English databases (PubMed, Web of Science, Cochrane Library) with retrieval time from database establishment to March 2025. Search keywords included: cardiac intensive care unit, CCU, specialized nurse, competency, training system, Delphi method, critical care nursing. Taking the PubMed as an example, a combination of free terms and subject headings was used for the search, with the search strategy as: (“Competency-Based Education”[Mesh] OR “Competency Based Education”[Ti/Ab] OR “Education, Competency-Based”[Ti/Ab] OR “Competency-Based Educations”[Ti/Ab] OR “Education, Competency Based”[Ti/Ab] OR “Educations, Competency-Based”[Ti/Ab]) AND (“Intensive Care Units”[Mesh] OR “Intensive Care Unit”[Ti/Ab] OR “Unit, Intensive Care”[Ti/Ab] OR “ICU”[Ti/Ab] OR “Intensive Care Units”[Ti/Ab]) AND (“Nurse Specialists”[Mesh] OR “Nurse Specialist”[Ti/Ab] OR “Specialist, Nurse”[Ti/Ab] OR “Specialists, Nurse”[Ti/Ab]). Inclusion criteria: studies focusing on ICU/CICU nurse competency evaluation, specialized nurse training framework construction, Delphi research on nursing indicator system. Exclusion criteria: review without original data, repetitive published literature, non-nursing population research, non-standardized training research. After screening, 28 publications were included. We extracted items on the dimensions and indicators of training system.

#### 2.2.2 Semi-structured qualitative interviews

Based on competency dimensions and indicators extracted from literature review, a semi-structured interview outline was compiled, covering expected collect their opinions on the content, structure, and implementation requirements of the training system for CICU specialized nurses. Purposive sampling was adopted to recruit 11 interviewees from three tertiary hospitals in Shanghai, including 2 CICU doctors, 2 CICU head nurses, 7 senior specialized nurses with ≥10 years CICU working experience. All interviews were carried out in independent quiet meeting rooms inside hospitals, each interview lasted 30–60 min with voice recording permitted after informed consent. Recordings were transcribed into text within 24 h; Colaizzi seven-step qualitative analysis was adopted to code, classify and summarize original viewpoints, extract competency items of the training system.

#### 2.2.3 Formation of initial version of competency-based CICU specialized nurse training indicator system

Integrating literature review and qualitative interview results, we formed the initial draft of CICU specialized nurse training indicator system, including 4 primary-level indicators, 19 secondary-level indicators and 60 tertiary-level indicators. The whole construction process strictly complied with the CREDES reporting specification for Delphi research (18, 19).

#### 2.2.4 Preparation of Delphi expert consultation questionnaire

The Delphi expert consultation questionnaire was designed on the basis of the established training system, including three main parts: an introduction to the study purpose and significance, expert basic information collection, and the draft version of a training system for CICU specialized nurses. Experts were asked to rate the importance of each indicator on a Likert 5-point scale (5 = extremely important, 4 = important, 3 = moderately important, 2 = slightly important, 1 = unimportant), and leave space for experts to put forward suggestions for modification or supplementation on each indicator. Before the formal consultation, a pre-investigation was conducted with 3 experts (majoring in CICU nursing and nursing education) to test the reasonableness of the questionnaire expression and structure, and the questionnaire was adjusted and improved according to the pre-investigation feedback to form the final version of Delphi consultation questionnaire.

### 2.3 Delphi consultation expert’s selection

Purposive sampling was adopted in this study for the Delphi process. The inclusion criteria for Delphi experts were as follows: ① Engaged in coronary intensive care medicine, coronary intensive care nursing, coronary intensive care nursing management, or nursing education for ≥10 years; ② Held a bachelor’s degree or higher; ③ Possessed an associate senior professional title or above; ④ Familiar with the training system for specialized critical care nurses and voluntarily participated in this consultation. From May to August 2025, the consultation questionnaires were distributed via email and WeChat surveys, and completed questionnaires were returned by the experts within 2 weeks.

### 2.4 Implementation of Delphi process

After each round of questionnaire recovery, the research team compiled descriptive statistical results, including the mean importance score, coefficient of variation (CV), and expert suggestions. All feedback from the first round of experts was consolidated and distributed to all experts along with the second round questionnaire for their reference during the re-scoring process. Indicators with a mean importance score greater than 3.50 and a CV less than 0.25 were directly retained (20). For items that did not meet both thresholds simultaneously, the research team summarized all experts’ consistent suggestions for modification, addition, or deletion, rather than arbitrarily removing items. All disputed items were preserved and returned to the entire expert panel for secondary scoring in the next round to avoid omitting clinically critical indicators. The interval between the two consultation rounds was 30 days. Expert opinions converged after two rounds without significant divergent suggestions, leading to the termination of the Delphi procedure.

### 2.5 Statistical analysis

Data entry and statistical analysis were performed using SPSS 27.0 and Excel software. Count data were expressed as frequency and percentage (%), measurement data were expressed as mean ± standard deviation, and expert participation degree was expressed by the questionnaire response rate (%). Expert judgment basis coefficient (Ca) and familiarity coefficient (Cs) were used to calculate authority coefficient Cr = (Ca + Cs)/2. Expert consensus level was reflected by indicator importance score, and coordination degree was measured by Kendall’s W and CV. The significance level for hypothesis testing was set at *α* = 0.05. Analytic hierarchy process was used to build hierarchical indicator structure, calculate weight of each level indicator and complete consistency check.

## 3 Results

### 3.1 General information of experts

A total of 23 experts from four provinces and municipalities participated in Delphi consultation. The basic information of the experts was as follows: the average age was 45.6 ± 5.2 years, and the average years of work experience was 20.3 ± 6.1 years. Among the participants, 8 experts held senior professional titles (34.79%), 8 held associate senior professional titles (34.79%), and 7 held titles of attending physician or chief nurse (30.42%). In terms of academic qualifications, 4 experts possessed doctoral degrees (13.39%), 11 held master’s degrees (47.83%), and 8 had bachelor’s degrees (34.78%). The areas of expertise among the experts included 4 in critical care medicine (17.39%), 8 in nursing management (37.48%), 8 in critical care nursing (37.48%), and 3 in nursing education (13.04%), as shown in Table 1.

**Table 1:** General information of experts (*N* = 23).

General Information	Classification	Number	Percentage (%)
Gender	Male	4	17.39
	Female	19	82.61
Professional Title	Medium grade	7	30.42
	Deputy senior title	8	34.79
	Senior title	8	34.79
Educational Background	Bachelor	8	34.78
	Master	11	47.83
	PhD.	4	13.39
Master/PhD. Tutor	Yes	13	56.52
	No	10	43.48
Professional field	CICU/ICU Doctor	4	17.39
	Nursing management	8	37.48
	CICU/ICU nurse	8	37.48
	Nursing Education	3	13.04

### 3.2 Expert participation coefficient

In both rounds of the Delphi survey, 23 questionnaires were distributed, with 23 valid responses collected, achieving a 100% effective response rate. In the first round, 10 experts (43.48%) provided 7 modification suggestions, while in the second round, expert opinions tended to converge, demonstrating a high level of engagement among the participants.

### 3.3 Expert authority and coordination degree

In this study, the first round of expert consultation yielded a Ca of 0.835 and a Cs of 0.933, with Cr being 0.884; the second round resulted in Ca = 0.852, Cs = 0.935, and Cr = 0.894. All values exceeded 0.8, indicating high expert authority. For the first round, Kendall’s W value was 0.241 and chi-square was 428.71 (*p* = 0.001); for the second round, W value was 0.247 and Chi-square was 457.30 (all p = 0.001). This suggests that expert opinions tended to converge, shown in Table 2.

**Table 2:** Results of statistical test of expert coordination coefficient.

Rounds	The number of experts	Kendalls W	X2	P
Round 1	23	0.241	428.71	0.001
Round s	23	0.247	457.30	0.001

### 3.4 Formation of competency-based CICU specialized nurse training indicator system

In the first round, 10 experts proposed 7 revision suggestions. In the second round, expert opinions tended to converge, with no further suggestions put forward. During the first round of expert consultation, based on expert feedback, 3 tertiary-level indicators were added and 4 tertiary-level indicators were modified. Experts recommended adding “clinical innovation practice competence,” “CICU patient comfort assessment and implementation principles,” and “pain assessment and management theory.” They also suggested revising “orotracheal cannula cooperation and airway management” to “tracheal intubation coordination and airway management,” “severe arrhythmia medication and electrical cardioversion cooperation” to “identification and emergency management of severe arrhythmias,” “condition assessment and risk prediction” to “predictive nursing and risk assessment,” and “infection decision-making for critically ill patients” to “standardized infection prevention and control strategies for critically ill patients.”

All expert recommendations were preserved and returned to the entire expert panel for secondary scoring in the second round, and no new suggestion was raised by experts. Then the final training indicator system was formed covering 4 primary, 19 secondary and 63 tertiary indicators. The weight coefficient and importance score of each hierarchical indicator are listed in Table 3.

**Table 3:** Importance assignment of training system for CICU specialist nurses.

Primary-level indicators	Secondary-level indicators	Tertiary-level indicators	Importance assignment(±s)	CV	Weight
Professional knowledge			4.91 ± 0.29	0.059	0.256
	Cardiovascular Critical Care Theory		4.70 ± 0.47	0.100	0.053
		Pathophysiology and key nursing considerations of Acute Myocardial Infarction	4.52 ± 0.59	0.131	0.015
		Severe heart failure classification and volume management	4.91 ± 0.29	0.059	0.017
		Diagnostic criteria and resuscitation strategies for cardiogenic Shock	4.91 ± 0.29	0.059	0.017
		Arrhythmia classification and emergency management	4.91 ± 0.29	0.059	0.017
	Cardiovascular pharmacotherapy		4.74 ± 0.45	0.095	0.053
		Vasoactive drug titration and adverse reaction monitoring	4.91 ± 0.29	0.059	0.017
		Specifications for antithrombotic drug use and bleeding risk assessment	4.74 ± 0.45	0.095	0.017
		Clinical application of diuretics and management of electrolyte balance	4.48 ± 0.59	0.132	0.015
		Positive muscle strength mechanism of drug action and nursing care	4.87 ± 0.34	0.070	0.017
	Theory of life support technology		4.91 ± 0.29	0.059	0.055
		Principles and parameter adjustment of mechanical ventilation	4.91 ± 0.29	0.059	0.017
		ECMO Principles of operation and complication prevention	4.74 ± 0.45	0.095	0.017
		IABP indications and key nursing points	4.52 ± 0.59	0.131	0.015
		PiCCO monitoring theory and Data interpretation	4.53 ± 0.51	0.114	0.015
	Comfort care and pain management		4.48 ± 0.59	0.114	0.050
		Core concepts of comfortcare	4.53 ± 0.51	0.131	0.015
		CICU patient comfort assessment and implementation principles	4.70 ± 0.47	0.100	0.016
		Pain assessment and management theory	4.70 ± 0.47	0.105	0.016
	Infection control and occupational protection		4.74 ± 0.45	0.095	0.053
		CICU hospital-acquired infection prevention and control standards	4.74 ± 0.45	0.095	0.016
		Multidrug-resistant organism management and occupational protection	4.74 ± 0.45	0.095	0.016
		Standardized infection prevention and control strategies for critically Ill patients	4.70 ± 0.47	0.100	0.016
Professional skills			4.57 ± 0.51	0.111	0.240
	Life support operations		4.61 ± 0.50	0.108	0.052
		Tracheal intubation coordination and airway management	4.65 ± 0.49	0.105	0.016
		Mechanical ventilation parameter settings and alarm handling	4.83 ± 0.39	0.080	0.016
		ECMO Circuit care and emergency management	4.52 ± 0.46	0.101	0.015
		Operation and maintenance of the IABP device	4.65 ± 0.49	0.105	0.016
	Monitoring of technical operations		4.52 ± 0.51	0.113	0.051
		PiCCO catheter insertion and data monitoring	4.83 ± 0.39	0.080	0.016
		Arterial blood gas analysis collection and result interpretation	4.70 ± 0.47	0.100	0.016
		Continuous cardiac output monitoring and reporting	4.87 ± 0.34	0.071	0.017
		Application of bedside ultrasound in circulatory assessment	4.78 ± 0.72	0.088	0.016
	Emergency Response Skills		4.74 ± 0.45	0.095	0.053
		First aid procedures for cardiac Arrest	4.78 ± 0.42	0.080	0.016
		Identification and management of acute cardiac tamponade	4.87 ± 0.34	0.071	0.017
		Identification and emergency management of severe arrhythmias	4.83 ± 0.39	0.080	0.016
	Comprehensive nursing care and pain management procedures		4.74 ± 0.45	0.095	0.053
		Practical application of comfortable nursing care	4.65 ± 0.49	0.105	0.016
		Posture management and prevention of pressure ulcers	4.65 ± 0.49	0.105	0.016
		Application of pain assessment tools and non-pharmacological analgesic techniques	4.52 ± 0.51	0.113	0.015
	Specialized Operational Skills		4.65 ± 0.49	0.105	0.052
		Nursing care for closed thoracic drainage	4.70 ± 0.47	0.100	0.016
		Emergency pacemaker care and parameter adjustment	4.78 ± 0.42	0.088	0.016
		Vascular access maintenance	4.78 ± 0.42	0.088	0.016
Professional competence			4.78 ± 0.42	0.088	0.252
	Professional ethics		4.65 ± 0.49	0.105	0.052
		Patient privacy protection and information security	4.57 ± 0.51	0.111	0.016
		Professional value identification in intensive care nursing	4.57 ± 0.51	0.111	0.016
		Ethics and techniques in doctor-patient communication	4.74 ± 0.45	0.095	0.016
	Psychological adjustment ability		4.65 ± 0.49	0.105	0.052
		Work stress management and emotional regulation	4.83 ± 0.39	0.080	0.016
		Psychological assessment and support for severe patients	4.78 ± 0.42	0.088	0.016
		Family psychological counseling and bereavement support	4.74 ± 0.45	0.095	0.016
	Teamwork		4.83 ± 0.39	0.080	0.054
		Communication and collaboration within a multidisciplinary team	4.74 ± 0.45	0.095	0.016
		Application of the SBAR standardized communication model	4.87 ± 0.34	0.070	0.017
		Team division of responsibilities and coordination in emergency situations	4.74 ± 0.45	0.095	0.016
	Lifelong learning		4.74 ± 0.45	0.095	0.053
		Evidence-Based nursing practice competence	4.61 ± 0.50	0.108	0.016
		Professional literature search and interpretation	4.61 ± 0.50	0.108	0.016
		Clinical innovation practice competence	4.70 ± 0.50	0.100	0.016
	Humanistic nursing competence		4.87 ± 0.34	0.070	0.055
		Humanistic communication skills	4.87 ± 0.34	0.070	0.017
		Cultural background-friendly nursing care	4.78 ± 0.42	0.088	0.016
		Humanistic care at the end of life	4.65 ± 0.49	0.105	0.016
		Resolution of cultural conflicts	4.70 ± 0.47	0.100	0.016
Practical competence			4.74 ± 0.45	0.095	0.250
	Clinical decision-making ability		4.74 ± 0.45	0.095	0.053
		Predictive nursing and risk assessment	4.78 ± 0.42	0.088	0.016
		Identification of nursing issues and adjustment of treatment plans	4.78 ± 0.42	0.088	0.016
		Development of care plans for complex cases	4.78 ± 0.42	0.088	0.016
	Teaching guidance skills		4.65 ± 0.49	0.105	0.052
		CICU new nurse training program	4.78 ± 0.42	0.088	0.016
		Organization of nursing rounds and case discussions	4.74 ± 0.45	0.095	0.016
		Health education for patients and their families	4.83 ± 0.39	0.080	0.016
	Quality improvement capability		4.74 ± 0.45	0.095	0.053
		Analysis and improvement of nursing adverse events	4.87 ± 0.34	0.070	0.017
		Monitoring of quality-sensitive indicators in nursing care	4.74 ± 0.45	0.095	0.016
		Application of the PDCA cycle in improving nursing quality	4.74 ± 0.45	0.095	0.016
	Continuity of care capability		4.61 ± 0.50	0.108	0.052
		Discharge plan development and follow-up management	4.61 ± 0.50	0.108	0.016
		Home rehabilitation guidance and complication prevention	4.70 ± 0.47	0.100	0.016

## 4 Discussion

### 4.1 Scientificalness of competency-based CICU specialized nurse training indicator system

The representativeness and authority of expert panel determine the reliability of Delphi-derived indicator system (21). This study strictly implemented standardized expert inclusion criteria, recruiting 23 specialists covering CICU medical treatment, clinical nursing, nursing management and nursing education across four regions, which avoided the single-source limitation of only recruiting clinical frontline nurses as seen in previous ICU competency research (13). The 100% questionnaire recovery rate, Cr>0.8 and gradually rising Kendall’s W across two rounds fully verified stable expert consensus, consistent with the Delphi methodological standard proposed by Jünger et al. (18) All indicators obtained importance scores over 4.47 with CV between 0.070–0.131, which was superior to the variation range of general ICU nurse training indicators reported by Wu et al. (13), reflecting stable expert recognition of all constructed modules.

We adopted AHP to calculate hierarchical indicator weights; primary indicator weights fluctuated within 0.240–0.256, secondary indicators 0.050–0.055 and tertiary indicators 0.015–0.017, showing balanced allocation without excessive bias toward theoretical knowledge or operational skills. Different from previous domestic research focusing only on general critical care competency (6, 10), this study integrated literature review and qualitative interview to capture CICU clinical demands, embedding cardiovascular exclusive technical modules into the indicator framework. The entire construction process follows the competency theory that aligns post requirements with training content, forming a complete theoretical framework ranging from dimension extraction and draft compilation to expert optimization. This framework provides solid methodological support for the subsequent implementation of clinical training and the evaluation of training effects.

The indicator system constructed in this study highlights the core position of professional competency specific to cardiovascular critical care, which fills the gap of targeted competency evaluation tools for CICU nurses in current research. At present, the rapid development of cardiovascular interventional therapy and critical care technology has put forward higher requirements for the professional ability of CICU nurses (22, 23), and the lack of standardized competency indicators has become a key bottleneck restricting the improvement of CICU nursing quality. The balanced weight distribution of each dimension in this study not only ensures the basic requirement of comprehensive competency cultivation, but also highlights the particularity of cardiovascular critical care work, which is more in line with the actual post needs of CICU nurses than the general ICU competency indicator system. This indicator system can provide a clear direction for the development of CICU nurse training courses, the formulation of competency assessment standards and the construction of talent echelon, and help to improve the pertinence and effectiveness of CICU nurse training.

### 4.2 Specialty characteristics and practical value of the training Indicator system

Most traditional critical care nurse training models prioritize knowledge infusion while ignoring comprehensive post competence cultivation, resulting in disjointed training and clinical practice (24). This study constructed a a training system, covering technical operations and core abilities such as interdisciplinary collaboration, psychological adjustment and evidence-based nursing quality improvement, which can effectively bridge the gap between training content and actual clinical needs, and address the common problem of incomplete ability coverage in current critical care nurse competency systems (25, 26).

Compared with existing domestic CCU training research (5), this training system integrates exclusive cardiovascular critical care modules such as ECMO nursing, PiCCO hemodynamic monitoring and individualized medication management as indicators. Meanwhile, standardized SBAR communication, multi-disciplinary team collaboration and humanistic hospice care modules are independently set up, responding to the high emergency and high emotional support demand of CICU clinical work, consistent with the humanistic nursing construction viewpoint of Jin et al. (24). The added clinical innovation practice dimension conforms to the trend of continuous renewal of cardiovascular critical care technology, guiding nurses to transform from single clinical operators to clinical practice improvers, which is rarely involved in prior domestic competency evaluation systems (12).

The integration of specialized technical ability, collaborative communication, psychological adjustment and humanistic care makes this system a standardized educational tool rather than a simple ability evaluation scale. Hospitals can decompose each tertiary indicator into targeted theoretical courses, simulated skill training and post assessment items, realizing the organic combination of competency evaluation and daily standardized nurse education.

### 4.3 Application recommendations for the competency-based training system of CCU specialist nurses

Combined with the structural characteristics of this multi-layer indicator system, three implementation suggestions are proposed for clinical nursing education management. First, adopt integrated theoretical and practical teaching mode matched with indicator classification. Theoretical knowledge modules such as circulatory support principle should be immediately followed by simulated operation training of tracheal intubation cooperation and ECMO complication management; high-risk emergency scenarios can use case-based group discussion to strengthen nurses’ clinical decision-making ability, which is consistent with the training optimization strategy of Awang et al. (16). Second, realize dynamic iterative adjustment of the training system (27). Nursing educators should regularly collect training feedback based on quality improvement indicators including adverse event analysis, supplement targeted training modules for weak competency links such as multi-drug resistant organism prevention, and adjust teaching forms to maintain the timeliness of the whole training framework. Third, attach importance to psychological and humanistic competency training (28). Set independent courses of occupational stress regulation, critical patient psychological assessment and terminal humanistic care according to corresponding tertiary indicators, alleviate CICU nurses’ professional burnout, and improve the humanistic temperature of cardiovascular critical care services.

## 5 Limitation

This study has several limitations. First, all experts included in this study are from clinical nursing fields in China, and the representativeness of experts from other regions or other nursing-related backgrounds has not been fully considered, which may affect the generalizability of the research conclusions to a certain extent. Second, the Delphi process relies on experts’ subjective judgment to a certain degree, and the possibility of deviation in individual expert opinions still cannot be completely eliminated. Third, this study only constructs the competency system, and subsequent verification and improvement through large-scale empirical applications are still needed to test the practical applicability of the system in different clinical nursing scenarios. Future research can expand the regional and disciplinary coverage of Delphi experts, carry out prospective empirical research in multiple CICU clinical settings to test the actual training effect of this indicator system, and continuously optimize hierarchical indicators to form a more universal national standard for CICU specialized nurse standardized training.

## 6 Conclusion

This study employs competency theory as its theoretical framework. We extracted the multi-dimensional competency demands specific to CICU specialized nurses through literature review and qualitative interviews. Ultimately, we constructed a multi-layer standardized training system for CICU specialized nurses via two rounds of Delphi expert consultation and AHP weight calculation. The training system established in this study covers core dimensions including professional knowledge, clinical skills, professional competencies and professional ethics, with clear weight distribution for each indicator. It can provide a scientific reference for the standardized training, competency assessment and professional development of CICU specialized nurses, and help to improve the overall quality of CICU nursing teams and the level of clinical nursing services. Future research can further expand the scope of expert consultation, verify the applicability of this indicator system in different levels of medical institutions, and continuously optimize the system based on practical application feedback to better adapt to the development needs of CICU specialized nursing.

## Data Availability

The raw data supporting the conclusions of this article will be made available by the authors, without undue reservation.
